# Augmentation of proximal femoral nail in unstable trochanteric fractures

**DOI:** 10.1051/sicotj/2016052

**Published:** 2017-02-13

**Authors:** Wasudeo M. Gadegone, Bhaskaran Shivashankar, Vijayanad Lokhande, Yogesh Salphale

**Affiliations:** 1 Department of Orthopaedic and Traumatology, Associate Professor of Orthopaedics, GMC 442402 Chandrapur Maharashtra India; 2 Iyer Orthopaedic Centre 103, Railway Lines 413001 Solapur Maharashtra India; 3 Department of Orthopaedic and Traumatology, Assistant Professor of Orthopaedics, Smt. Kashibai Nawale Medical college Maharashtra India

**Keywords:** Augmentation, Additional screw, Proximal femoral nail, Lateral trochanteric wall, Unstable trochanteric fracture

## Abstract

*Introduction*: Biomechanically proximal femoral nail (PFN) is a better choice of implant, still it is associated with screw breakage, cut out of screw through femoral head, Z effect, reverse Z effect, and lateral migration of screws. The purpose of this study is to evaluate the results of augmented PFN in terms of prevention of postoperative complications and failure rates in unstable trochanteric fractures.

*Material and methods*: We carried out a prospective study of 82 cases with unstable trochanteric femoral fractures from April 2010 to December 2015. Forty-two females and 40 males in the age group between 58 and 81 years were included in this study. There were 45 cases of AO 31 A2 (2.2, 2.3) and 37 cases of AO 31 A3 (3.1, 3.2, 3.3). Fractures were fixed by PFN with augmentation by an additional screw from trochanter to inferior quadrant of femoral head or cerclage wire to strengthen the lateral trochanteric wall.

*Results*: The bone healing is observed in all the cases in the mean period of 14.2 weeks. Nine patients developed complications, including lateral migration of neck screws (*n* = 5), Z effect (*n* = 1), infection (*n* = 2), and breakage of distal interlocking bolt in one case. Removal of screws was required in five cases. Patients were followed up for a mean of 8.4 months. At the end of follow-up the Salvati and Wilson hip function was 32 (out of 40) in 88% of patients.

*Conclusion*: The stabilization of lateral trochanteric wall with additional screw or cerclage wire increases the stability of construct.

## Introduction

Unstable trochanteric fractures continue to be a challenge for orthopedic surgeons. Despite high union rates, the functional outcomes still tend to be disappointing. Use of sliding hip screw in unstable trochanteric fractures is associated with significant medial displacement of the shaft resulting from excessive sliding of screw within the barrel and a higher incidence of screw cut-out [1, 2]. Intact lateral wall plays a key role in stabilization of unstable trochanteric fractures by providing a lateral buttress for proximal fragment, and its deficiency leads to excessive collapse and varus malpositioning [3]. Dynamic hip screw (DHS) with trochanteric buttress plate stabilizes the trochanteric fracture but at the cost of open procedure with significant blood loss [4]. The locking plate technology coupled with built-in metaphyseal contour enables fixation using the minimally invasive plate osteosynthesis (MIPO) technique, but the literature describes a high complication rate [5, 6].

Intramedullary nailing has become a popular method of stabilization of unstable trochanteric fractures in adults [7, 8]. Biomechanically it is a better choice of implant for fixation of unstable fractures as nail itself gives support to posteromedial wall and resists excessive collapse [9, 10]. Near-anatomical reduction and optimal positioning of implants are of paramount importance for good outcome and reducing the risk of complications. Still there are some pitfalls as implant failure does occur in PFN due to specific unbalanced biomechanical forces acting on implant around hip joint. A common complication of the PFN surgery is implant failure, which can be due to backout of screws, cut through of implant through bone, “Z” effect, and “reverse Z” effect or breakage of implant [11].

The objective of our study is to hypothesize that anatomical reduction and supporting the lateral wall is important to prevent complications. Augmentation of PFN with an additional screw through the greater trochanter or cerclage wire stabilizes the anatomically reduced greater trochanter, reducing the size of the medullary canal and increasing the stiffness of the bone-implant construct reducing the rate of complications.

## Methods

A prospective, nonrandomized study was conducted from April 2010 to December 2015. Eighty-two patients of unstable trochanteric fractures available for study with a mean age of 66 years (range: 58–81 years) with 42 females and 40 males were included. The AO/OTA classification was used to classify the fractures (A2, 2-3 = 45, A3, 1-2-3 = 37) (Table 1). Fall from standing height was the most common mode of injury, accounting for 85% of the cases, with the remainder sustaining injury in road traffic accidents.

**Table 1.** Demographic characteristics of the sample (*n* = 82).

	
Number of patients	82
Age	58–81 years
Females	42
Males	40
A2	45
A3	37
AO classification	82

Proximal femoral nail (nail length: 180 mm for A2 fractures, 250 mm and long PFN for A3 fractures) having a proximal diameter of 15 mm, with 8 mm compression and 6.4 mm derotation screw, was used. Distal locking was carried out with a 4.9 mm bolt. The nail has a 6° medio-lateral angle for easy insertion and tapered distal tip to avoid stress generation [10].

**Figure 1.**
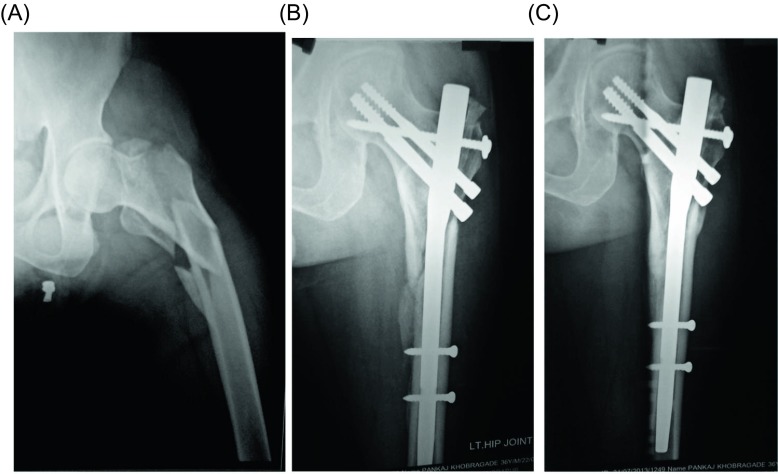
A 72-year-old female patient with a type 31-A3.3 fracture. (A) Preoperative Initial radiograph. (B) Radiograph of the proximal femur at the 6th postoperative week with additional screw with washer. (C) Radiograph of the proximal femur at the 12th postoperative month showing good consolidation of fracture.

Average delay from time of injury to fixation was three days (range: 2–12 days), which was mostly due to delay in reporting to the hospital. After appropriate anesthesia, the patients were placed in a supine position over the traction table. Fractures were reduced by closed means in most of the cases. Seventeen cases required mini open reduction to achieve anatomical or near-anatomical reduction. Schanz screws, Steinman pin, and bone hook were used as a joystick to achieve reduction in desired position. Percutaneous reduction clamps were used to maintain the reduction. In burst fracture, trochanteric fragments were clamped with towel clip and reduction clamps. Two 3 mm Kirschner wires were passed from greater trochanter to lower part of femoral head to fix the fracture temporarily preventing loss of reduction during insertion of the nail. The actual reduction of fragments can be seen in fluoroscopy while the reamer passes across the Steinman pin which is subsequently replaced by Poller (additional screw) screw. A 5-cm skin incision was initially made from the cranial part of the greater trochanter, and a guide wire was passed through the medial part of the tip of the greater trochanter distally, followed by trochanteric reaming over the guide wire. Implantation of proximal femoral nail was done in standard fashion with proper placement of screws keeping the tip-apex distance (TAD) within 20 mm. In the four-part unstable trochanteric fractures, the main comminution lies at the posterior aspect. This results in a cavity or void at the posterior aspect of the trochanteric region which was noted in most of these cases. Under image intensification, the greater trochanteric fragments were reduced anatomically. With a stab incision, the Steinman pin was removed and subsequently replaced by Poller (additional screw) screw fixed, using cancellous screws with washer depending on the bone quality or fracture configuration (Figure 1).

**Figure 2.**
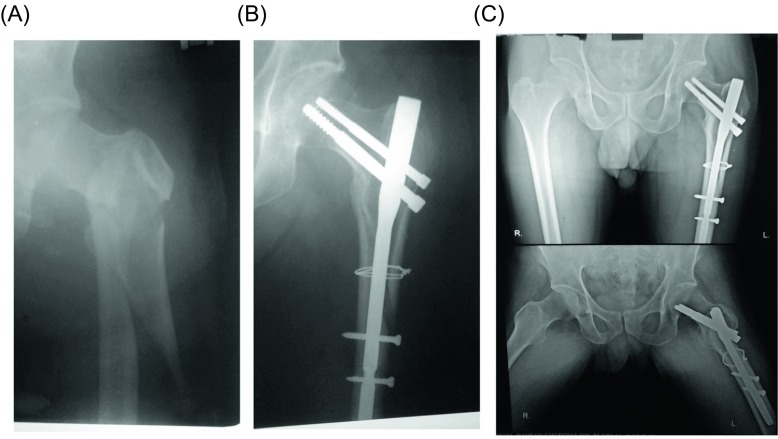
A 70-year-old female. (A) The initial preoperative radiographs. (B) Radiograph six weeks postoperatively with cerclage wire. (C) Anteroposterior radiograph at eight months postoperatively demonstrating the fracture healing.

In a reverse oblique fracture, the lateral wall was stabilized by introducing a screw at the lower pole of trochanter. The decision to add a cercumferential wire was often made in spiral or oblique fractures (Figure 2). The circumferential wire was inserted through prolonging the incision of compression screw on the lateral side of the thigh. Prolongation of about 1 cm incision is required in those cases where application of cerclage wire is done with minimum incision method with the help of wire passer. Therefore, there is minimal loss of blood and hence rates of post-operative blood transfusion incidence are not related to intraoperative procedure. In five cases of reverse oblique fractures, cerclage wire was used below the proximal locking screws. The tip-apex distance of the femoral neck screw within 20 mm was achieved in all the patients. Intraoperative details like operative time, blood loss, and number of blood units transfused were recorded.

The patients were encouraged to sit in bed and perform static exercises with the affected limb on the same day of operation. An X-ray examination was performed on the second postoperative day. At around 10th day postoperatively, the stitches were removed. Touchdown weight bearing with the help of a walker or crutches began two weeks after the surgery. One month after the surgery, progressive weight bearing was encouraged as tolerated. Full weight bearing was encouraged three months after surgery, based on the evidence of stability of construct and callus formation on radiographs.

Patients were followed up clinically and radiologically at regular intervals to look for progress of union and possible complications. All patients were followed up for a minimum period of seven months or till the fracture united. Clinical outcome was rated as per the Salvati and Wilson scoring system [12] at the time of final follow-up. Final radiological evaluation included any nonunion, malunion, screw cut-out, implant breakage, avascular necrosis of femoral head, and excessive sliding of screws.

## Results

The average follow-up period of our study was 8.4 months (average seven months to 16 months). Postoperative X-ray examination showed anatomical reduction in 78 cases, and acceptable reduction in four cases. Clinico-radiological consolidation of the fracture was observed in all cases at an average of 14.2 weeks (12–18 weeks). Mean duration of surgery was 65 min (45–80 min) in all the patients. Mean intraoperative blood loss was 80 mL (50–120 mL) and mean postoperative drainage in first 48 h was 70 mL. Patients with less than 10 gm% of hemoglobin received blood transfusion. In our series, 66 patients had hemoglobin between 8 and 10 gm, therefore transfusion was required in the majority of cases.

**Figure 3.**
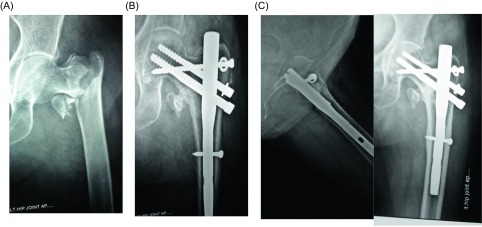
A 65-year-old male patient with a type 31-A3.3 fracture. (A) Initial radiograph. (B) Radiograph of the proximal femur at the 6th postoperative week with additional screw with washer. (C) Radiograph of the proximal femur at the 24th postoperative month showing complete consolidation of fracture.

**Figure 4.**
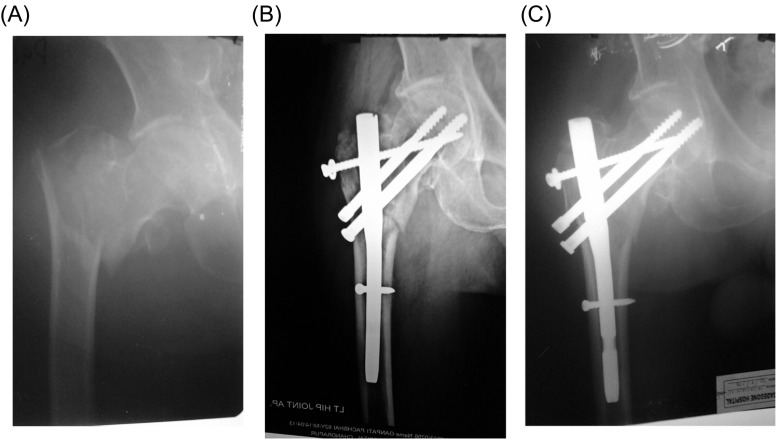
A 75-year-old male. (A) The initial preoperative radiographs. (B) Radiograph six weeks postoperatively with additional screw with washer. (C) Anteroposterior radiograph at 10 months postoperatively demonstrating the fracture healing.

There were some local as well as some systemic complications (Table 1). Nine patients developed local complications including lateral migration of neck screws (*n* = 5), “Z” effect (*n* = 1), infection (*n* = 2), and breakage of distal interlocking bolt in one case. No case of nonunion or implant breakage was observed (Table 2). Three of the patients complained of persistent pain in the hip region because of impingement of the proximal screw which was scheduled for hardware removal. Two patients had moderate persistent pain due to varus malunion. The average sliding of the PFN in this study was observed to be 2.8 mm (2–5 mm). No limb length discrepancy was observed in any of our cases with anatomical reduction. Four cases had less than anatomical reduction observed in the immediate postoperative period resulting in 6–7 mm of shortening, but none of these cases required a shoe raise. Identifiable rotation of the proximal fragment on X-rays was not observed in any of our cases. Reoperation for removal of lag screws due to technical failure was required only in five cases after union of fracture six months postoperatively (Figures 3 and 4).

**Table 2.** Complications.

Complications	No. of patients
Systemic	
Chest infection	5
Urinary tract infection	4
Myocardial infarction	1
Local complications	
Superficial infection	2
Lateral screw migration	5
Z-effect	1
Breakage of interlocking bolt	1

The average score for pain according to Salvati and Wilson criteria was 8.6 out of 10. Normal walking was resumed in 68 patients, six patients needed a walking aid for long distances, and the remaining eight patients required a walking aid even for short distances. Preinjury function was regained in 68 patients, while very little restriction was observed in six patients and the remaining eight patients had restricted normal activities but were able to do most of the housework. The Salvati and Wilson score for overall hip function was >30 points in 68 patients and >20 points in the remaining 14 patients. At the end of follow-up the Salvati and Wilson hip function was 32 (out of 40) in 88% of patients.

## Discussion

Sliding screw can be considered the “gold standard” for treating stable trochanteric fractures, but excessive collapse with shortening and high failure rates are concerns about their use in unstable trochanteric fractures [1, 13]. In unstable trochanteric fractures, intramedullary devices have an advantage of load sharing with smaller bending moments allowing early weight bearing and preventing excessive collapse [14]. For unstable fractures, intramedullary implants generally present biomechanical advantages over their extramedullary counterparts [15, 16] and numerous studies have demonstrated satisfactory results following the use of such implants. Various intramedullary devices such as gamma nail, proximal femoral nail, proximal femoral nail antirotation, and Intertan integrated nail have been used for fixation of these fractures. The intramedullary proximal femoral nail was devised by the AO/ASIF group in 1996 with two proximal screws including an antirotation screw with the aim of increasing the stability of the fracture fixation. However, important complications include lateral protrusion of screws, cut through of screws, Z or reverse Z effect, and fracture of lateral trochanteric wall [17].

Traditionally, the medial and posteromedial fracture fragments have been considered to be important elements in determining the severity of intertrochanteric hip fracture [18]. However, preoperative or intraoperative fracture of the lateral femoral wall in addition to posteromedial void increases the instability and is an important predictor for reoperation in DHS. Recent workers stated that the lateral femoral wall was found to be the main predictor for a reoperation after an intertrochanteric fracture [3, 19, 20]. Surgeon’s experience and accuracy in performing the procedure is of great importance in preventing implant failure. We are of the opinion that lateral wall instability is also important in PFN surgery as it is in DHS fixation. Though intramedullary nailing is favored in the recent literature, failure rate of gamma nail for the treatment of these fractures ranges from 12.7% to 15% [21, 22]. Fogagnolo et al. showed a complication rate of about 23.4% with the use of PFN for the treatment of these unstable fractures [23]. In another study done by Uzun et al. [24] nonunion was seen in 5.7%, secondary varus collapse in 25.7%, cut out of proximal screws in 5.7%, and reoperation in 14.3% cases. Use of antirotation (PFNA) in the treatment of these unstable intertrochanteric fractures is also controversial with varying results, though it has some theoretical advantage over the DHS. Various authors have shown high complication rate with the use of these implants. As for PFNA, Takigami et al. [25] showed complications in 14% of the cases and 4% required reoperation. The proximal femoral nail compensates for a posteromedial defect acting as a buttress to prevent medialization but fails to provide stability on the lateral side if the lateral wall is compromised. Compromising the lateral wall leads to a situation in which internal strength of the bone of the head of the femur only will resist the medial deforming forces. All cases of implant failure show varus collapse invariably as the lateral wall fails to provide enough support to the implant. If we modify the operative procedure or implant design, being able to fix lateral wall support then may decrease implant failure. To prevent varus collapse, protecting forces (Abductor muscle force + internal strength of implant and bone) must be equal to or be more than deforming forces. Hence restoration of the lateral wall is of paramount importance to prevent varus collapse and further complications.

In unstable intertrochanteric fractures, the integrity of the lateral femoral wall can be restored with augmentation of PFN with an additional screw or cerclage wire to prevent complications. Improved bony contact between proximal and distal fragments by stabilization of the comminuted lateral wall is likely to improve the chances of union and maintenance of adequate lever arm. In our study, varus angulation of more than 5° was noted in six patients and Z effect in one patient. This complication may have occurred due to a technical problem where the screw was not getting enough purchase in the femoral head and lateral wall causing gradual loosening and protrusion. No case of screw cut-out was noted in our series as compared in the literature where cut out of screw is reported up to 2.5%. In five cases, lateral migration of screws was observed, most likely because of the shearing forces caused by the tendency for lateral displacement of the proximal end and medial displacement of the distal end in unstable fractures. The average sliding of the DHS in the study of Gupta et al. [3] was observed to be 3.4 mm in unstable fractures treated by DHS with trochanteric stabilization plate. In our study the average sliding of the screw was found to be 2.8 mm. Babst et al. [4] also reported significant reduction in excessive collapse and subsequently reduced limb length discrepancy by using a trochanter stabilizing plate (TSP) in combination with the DHS, but it is an invasive method with significant blood loss in comparison with our minimally invasive method with significant superior results. Excessive fracture collapse results in shortening of the abductor lever arm leading to permanent limping and increases morbidity. In our study, only five patients who had >15% of fracture collapse scored “Fair” at 24 weeks. This shows the importance of preventing excessive fracture collapse in order to improve the final clinical outcome. In cases with severe osteoporosis that interferes with proximal screw fixation, screws augmented with cement may be used to increase stability as suggested by Alexa and Cozma [26]. In areas of cement augmentation, an additional screw effectively prevents the rotation of the proximal fragment. Therefore in our series, reoperation rate for removal of screws was only 4.1%. Healing time was 14.2 weeks. The functional results in this study were graded as excellent in 74% of the cases and good in 26% of the cases according to the Salvati and Wilson scoring system.

In our series, complications are fewer when compared to the technical and mechanical complications described in the literature [21–24].

The lateral wall reconstruction significantly decreased the incidence of lateralization of the greater trochanter with limited telescoping of comminuted fragments following weight bearing. These corrective measures resulted in better hip abductor function and final Salvati-Wilson functional score with restoration of prefracture mobility. The shortcoming of our study is that the mechanism of action of the PFN with an additional screw has not been evaluated in biomechanical studies. Further biomechanical and clinical studies are necessary to validate the efficacy of PFN augmentation.

## Conclusion

Augmentation of PFN with an additional screw is simple yet useful technique in the treatment of unstable trochanteric fractures ensuring significant reduction in excessive collapse and subsequently reduced limb length discrepancy. It creates a biomechanically stable construct and overall superior functional and radiological outcomes in patients with unstable trochanteric fractures.

## Conflict of interest

The author declares no conflict of interest in relation with this paper.
